# Etiology of the Degeneracy of the Human Teeth

**Published:** 1901-12-15

**Authors:** R. B. Adair


### ETIOLOGY OF THE DEGENERACY OF THE HUMAN TEETH.

BY DR. R. B. ADAIR.

Read before the Georgia State Dental Society.

The etiology of the degeneracy of the human teeth was enthusiastically discussed by the dental profession a few years ago. Our association proceedings and journals were full of interesting discussions upon this subject.

The accepted theory of our leading men at that time was to provide the proper kind of food for mothers during pregnancy and lactation, with the view that a larger amount be assimilated and turned into bone and teeth.

But about this time some prominent men of the profession, such as Dr. W. H. Morgan, of Nashville, proved by analysis that even rice, while the most barren of bone and tooth building materials, contained more lime salts than was possible for the system to assimilate. The profession dropped the discussion of the vital question, and it has not yet been revived.

Dr. Morgan and others have never convinced me to his theory, and I still maintain that if we would have well developed teeth we must lay aside our advanced ideas of living and provide the proper food for mothers during gestation and lactation.

During gestation the phosphates and carbonates are usually insufficient for the demands of the mother, and every day we may view the results by the softening and decay of the mother's teeth during this period. At no time of life are the proper foods so urgently needed as when two lives—one in embryo—have to live and be supplied through one channel.

By a study of the domestic animals we may learn valuable lessons as to the need of certain foods by the mother. If we keep lime from fowls they will lay soft eggs without shells. Cows grazed on land sown with bone phosphates will give richer milk. We also know that wheat will die soon after it germinates and blossoms will not come on peas that are planted on earth deprived of phosphates.

The debility from which so many women suffer during pregnancy is due to this lack of food that nature intended she should have. We see women during pregnancy craving lime salts to such an extent that they will eat chalk or even slate pencils.

When we consider that nature is at work long before birth constructing the second permanent set of teeth, the enamel organs of the first permanent molars appearing about the fifth month before birth, and the second molars three months after birth; that a child has from the eruption of its first molars forty-eight developing teeth in the mouth, and that the structural condition of all these depends upon its embryonic life, we should not fail to realize the vast importance of this vital question.

My brother, Dr. E. F. Adair, of Harmony Grove, Ga., was invited to read a paper upon dental hygiene before the Medical Society, which met in Augusta last month. I was told by those present that the paper was greatly enjoyed and did much good. I wish to give you an illustration from his experiments which will well illustrate the point I wish to make by this paper. In his county there was published and widely distributed a book upon Tocology, or so-called "child-birth made easy." The book called for a diet composed of nonbone and tooth elements for pregnant women, which made the bones soft and yielding, making child-birth easy. He observed twenty-two cases of children born under this process, all of them under two years old, and not in a single case did the child have any whole teeth. Those that did have teeth were suffering untold agony because the teeth were broken off at the gum line and great quantities of pus coming from around the teeth. All this evil was traced to the same cause. The mothers had been fed upon sweetmeats and delicacies containing starch, sugar, and fine bolted flour; in fact, depriving the mother of every single article that contained bone-building material.

When my brother married he found that his wife possessed teeth of very inferior character, and so were his, so he determined to carry out his idea of oral hygiene diet. He began with his first-born, but as this was his first experiment he did not fully carry out his idea, but even as far as he did go the result was gratifying. With his second child he failed to carry out his idea on account of his wife's sickness, and the result was so bad that he decided to force a strictly bone-building diet during the pregnancy of his next child. The result produced the best developed child he or any of the physicians ever saw. I have often seen the teeth of the first child. She is about twelve years of age, and her teeth are so perfect that they look like pearls, and not a speck of decay ever appeared on any of them. The third one is now fifteen months old and does not know the taste of medicine, not even a drop of oil. She is two months in advance with the eruption of her temporary teeth, and that without the slightest inconvenience or pain whatever.

The advance of civilization has done much to separate us from the animal, but it has likewise done much to produce degeneracy of the human teeth, and unless we do all in our power to correct this error, and require our patients who are about to become mothers to eat bone and tooth-building diet, we will see children with defective development, nearly edentulous. Then a whole army of dentists could not preserve their teeth.

Let us do our duty as professional men and exert our every energy to correct this great evil that threatens to curse our nation as a people. Let us recommend and require that the mothers who come under our charge carry out this oral hygiene diet during pregnancy and lactation.

CHEMICAL ANALYSIS OF DENTIN:

| | |
|---|---|
| Organic matter | 19.64 |
| Fat | .40 |
| Calcium Phosphate | 66.72 |
| Magnesium Phosphate | 1.18 |
| Calcium Carbonate | 3.62 |
| Other Salts | .83 |

ENAMEL:

| | |
|---|---|
| Calcium Phosphate | 89.82 |
| Calcium Carbonate | 4.37 |
| Magnesium Phosphate | 1.34 |

WHEAT, WHEN DRIED, CONTAINS:

| | |
|---|---|
| Glutine | 19.64 |
| Water | 14.83 |
| Albumen | .95 |
| Starch | 45.99 |
| Gum | 1.52 |
| Sugar | .87 |
| Fiber | 12.34 |
| Ash | 2.36 |

ASH OF WHEAT IS RICH IN MAGNESIUM AND POTASH. ITS COMPOSITION IS:

| | |
|---|---|
| Potash | 29.97 |
| Soda | 3.90 |
| Magnesia | 12.30 |
| Lime | 3.40 |
| Phosphoric Acid | 46.00 |
| Sulphuric Acid | .33 |
| | 2.53 |
| | .79 |
| Sodium Chloride | .09 |

As bread is the staff of life, let us begin by recommending the use of bread made from the whole grain of wheat. It is sweeter and more palateable, and contains just the approximate principles we desire for this diet. One way is to buy wheat, wash and dry it in the sun, then have the grain ground in a water mill like corn meal. Such cereals as oat meal, barley meal and rye meal would all make bread that is rich in lime and phosphates. Peas, beans and meats are all good bone food. The ends of leg bones of chickens should be prescribed. They are soft and women like them. Ground up, these bones make a fine soup which, if thickened with barley, is very rich in tooth-building ingredients.

Since preparing this paper I notice two very interesting articles in the May number of *Cosmos* bearing on this subject: one by Dr. Edward C. Kirk on the "Management of Infants' Mouths," which brings out clearly not only this idea of the cause of defective development of the human teeth, but another and very important cause of infantile colic, which every dentist and physician should read and then preach to his patients who are mothers.

The other article to which I refer was read at the Northeastern Dental Association, of Rhode Island, by Prof. H. D. Perky, President of the Oread Institution, Worcester, Mass., on "Nutrition as a Toothbuilder." This article is written not by a dentist but by a scientific man, who has taken the trouble to investigate this question, and who has given us the most scientific and at the same time most practical paper I have ever seen on this subject. I mention this simply to show that this great question of diet is beginning to be resurrected again and discussed by our leading journals and at our society meetings.—*Dental World.*

